# The complete mitochondrial genome and phylogenetic analysis of *Sinocyclocheilus angularis* (Cypriniformes: Cyprinidae)

**DOI:** 10.1080/23802359.2021.1920862

**Published:** 2021-11-18

**Authors:** Qi Luo, Renyi Zhang

**Affiliations:** School of Life Sciences, Guizhou Normal University, Guiyang, China

**Keywords:** *Sinocyclocheilus angularis*, mitochondrial genome, phylogeny

## Abstract

This is the first study to determine the complete mitochondrial genome of *Sinocyclocheilus angularis*, which is an endemic cavefish native to the Karst region of the Yunnan-Guizhou Plateau. The complete mitochondrial genome size of *S. angularis* was 16,586 bp, and it consisted of a control region, 13 protein-coding genes, 22 transfer RNA genes, and 2 ribosomal RNA genes. The base composition of the mitogenome was 25.14% A, 32.45% T, 27.10% G, and 15.31% C, with an overall GC content of 42.42%. Phylogenetic analysis revealed that all *Sinocyclocheilus* were clustered into one strong clade and *S. angularis* was very closely related to *S. bicornutus*. It will provide an essential genetic tool for the further evolutionary studies and conservation of genus *Sinocyclocheilus*.

*Sinocyclocheilus angularis*, which belongs to order Cyprinoidea, family Cyprinidae, genus *Sinocyclocheilus*, is an endemic cavefish native to the Karst region of the Yunnan-Guizhou Plateau (Zhao and Zhang 2009). In our study, we analyzed the complete mitochondrial genome sequence and phylogenetic relationship of *S. angularis*, to provide an essential genetic tool for the further evolutionary studies and conservation of genus *Sinocyclocheilus*.

In this study, *S. angularis* sample was collected from Pan county of Guizhou Province, Southwestern China (25°24.50′N, 104°43.29′E). The voucher specimen was stored in 99.5% ethanol and deposited in the fish specimen room, School of Life Science, Guizhou Normal University (Renyi Zhang, e-mail: zhangrenyi@gznu.edu.cn) under the voucher number GZNU202001332. Total genomic DNA was extracted from muscle using DNeasy Blood & Tissue Kit (QIAGEN, Hilden, Germany). The complete mitogenome DNA was sequenced on Illumina HiSeq platform (Illumina, San Diego, CA), and then the short raw sequences were assembled with MitoZ (Meng et al. 2019). The MitoAnnotator on MitoFish homepage was used for mitochondrial genome annotation (Iwasaki et al. 2013) and the web page of tRNAscan-SE was used for the transfer RNA (tRNA) genes identification (Lowe and Eddy 1997). The mitochondrial genome was submitted into GenBank with an accession number MW362289.

The complete mitochondrial genome size of *S. angularis* was 16,586 bp, which contained of a control region (D-loop), 13 protein-coding genes (PCGs), 22 transfer RNA (tRNA) genes, and 2 ribosomal RNA (rRNA) genes. The base composition of the mitogenome was 25.14% A, 32.45% T, 27.10% G, 15.31% C, and the overall GC content was 42.42%. The arrangement of these genes is similar to that other species of *Sinocyclocheilus* (Zhang and Wang 2018; Xu et al. 2019; Luo et al. 2019). All of genes were encoded on heavy strand (H-strand) or light strand (L-strand). Eight tRNAs (*tRNA-Gln*, *tRNA-Ala*, *tRNA-Asn*, *tRNA-Cys*, *tRNA-Tyr*, *tRNA-Ser*, *tRNA-Glu*, *tRNA-Pro*) and *ND6* gene were encoded on the L-strand and the other genes (*tRNA-Phe*, *12S rRNA*, *tRNA-Val*, *16S rRNA*, *tRNA-Leu*, *ND1*, *tRNA-Ile*, *tRNA-Met*, *ND2*, *tRNA-Trp*, *COI*, *tRNA-Asp*, *COII*, *tRNA-Lys*, *ATPase 8*, *ATPase 6*, *COIII*, *tRNA-Gly*, *ND3*, *tRNA-Arg*, *ND4L*, *ND4*, *tRNA-His*, *tRNA-Ser*, *tRNA-Leu*, *ND5*, *Cyt b*, *tRNA-Thr*) were encoded on the H-strand. All of PCGs began with ATG codon, except *COI* gene, which started with GTG. Seven PCGs ended with complete stop codon (TAA or TAG), but six genes (*ND2*, *COII*, *COIII*, *ND3*, *ND4*, *Cyt b*) stopped with incomplete (T- or TA-) codon.

To understand the evolutional relationship between *S. angularis* and other *Sinocyclocheilus*, we constructed a phylogenetic tree from 16 species of *Sinocyclocheilus* and 2 outgroup species (*Cyprinus carpio* and *Barbus barbus*). The phylogenetic tree based on 13 protein-coding genes of 18 species from Cyprinidae was established by using MrBayes (Ronquist and Huelsenbeck 2003) module in PhyloSuit (Zhang et al. 2020). The result of phylogenetic tree showed that all *Sinocyclocheilus* were clustered into one strong clade (Figure 1). The *S. angularis* was very close related to *S. bicornutus*, and the two species (*S. angularis* and *S. bicornutus*) and *S. altishoulderus* as a sister clade formed a monophyletic group with *S. furcodorsalis*.

**Figure 1.**
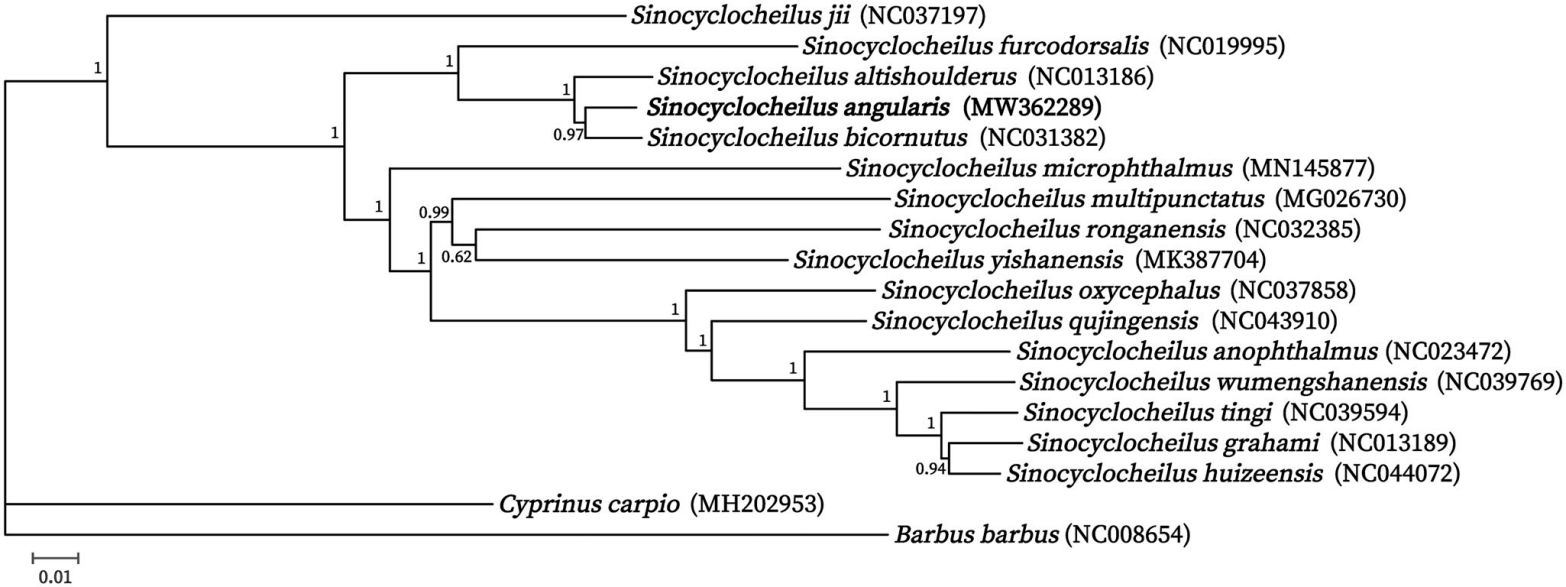
Phylogenetic relationship of *S. angularis* using Bayesian method by PhyloSuite. *Cyprinus carpio* and *Barbus barbus* were served as outgroups. The values at the internode branches represent Bayesian posterior probability.

## Data Availability

The data that support the findings of this study are openly available in NCBI at https://www.ncbi.nlm.nih.gov/nuccore/MW362289, reference number MW362289.
